# Pulsatile tissue deformation dynamics of the murine retina and choroid mapped by 4D optical coherence tomography

**DOI:** 10.1364/BOE.445093

**Published:** 2022-01-07

**Authors:** Bernhard Baumann, Conrad W. Merkle, Marco Augustin, Martin Glösmann, Gerhard Garhöfer

**Affiliations:** 1 Center for Medical Physics and Biomedical Engineering, Medical University of Vienna, Währinger Gürtel 18-20, 1090 Vienna, Austria; 2 Core Facility for Research and Technology, University of Veterinary Medicine Vienna, Veterinärplatz 1, 1210 Vienna, Austria; 3 Department of Clinical Pharmacology, Medical University of Vienna, Währinger Gürtel 18-20, 1090 Vienna, Austria

## Abstract

Irregular ocular pulsatility and altered mechanical tissue properties are associated with some of the most sight-threatening eye diseases. Here we present 4D optical coherence tomography (OCT) for the quantitative assessment and depth-resolved mapping of pulsatile dynamics in the murine retina and choroid. Through a pixel-wise analysis of phase changes of the complex OCT signal, we reveal spatiotemporal displacement characteristics across repeated frame acquisitions. We demonstrate in vivo fundus elastography (FUEL) imaging in wildtype mouse retinas and in a mouse model of retinal neovascularization and uncover subtle structural deformations related to ocular pulsation. Our data in mouse eyes hold promise for a powerful retinal elastography technique that may enable a new paradigm of OCT-based measurements and image contrast.

## 1. Introduction

The ocular in- and outflow of blood induces volumetric changes to the eye ball. It thus causes movement of ocular structures and a variation of the intraocular pressure synchronous with the cardiac cycle [1]. The relation of ocular pulsatility and biomechanical properties of eye structures such as elasticity and thus stiffness and rigidity have been investigated in preclinical and clinical studies [2–10]. Irregularities of ocular pulsatility have been associated with some of the most frequent and sight-threatening eye diseases including age-related macular degeneration (AMD) [11] and glaucoma [12,13]. While clinical studies are limited to the actual physiological and pathological states of volunteers and patients, preclinical studies in animal models have enabled the investigation of an extended, controlled range of scenarios, such as disease evolution and novel treatment avenues, on a fast-forward time scale spanning only a few months.

Measurements of ocular pulsatility have been performed based on pressure variations and relative tissue motion. Using tonometers to detect pulsatile changes of the intraocular pressure, first approaches such as pneumotonometry, modified Goldmann applanation tonometry, and dynamic contour tonometry were demonstrated [14–18]. These techniques assessed ocular pulsatility globally and without spatially resolved information or only indirectly via the ocular pulse amplitude. In contrast, optical techniques based on laser interferometry enabled pointwise measurements of displacements of one ocular structure (e.g., the retina) with respect to another reference tissue (e.g., the cornea) [19–21]. Optical fundus pulsation measurements have been shown to be a useful contact-free method for the assessment of a variety of physiological conditions, for studying medication-induced effects and for investigating diseased eyes. Still, fundus pulsations depend on location within the eye and most optical approaches only provided information on the pulsatile character at a single spatial location.

Optical coherence tomography (OCT) was introduced three decades ago as a noninvasive modality for imaging transparent and translucent samples and tissues [22]. By scanning a light beam over the tissue and measuring the echo time delay and intensity of backscattered light using low-coherence interferometry, OCT can perform micron scale imaging of biological tissues in situ and in real time [23,24]. In ophthalmology, OCT imaging enables the visualization of retinal structure and pathology with resolutions that are impossible to obtain with any other noninvasive technique [25]. During the last two decades, OCT has become a clinical standard for diagnosis and management of ocular diseases. State-of-the art clinical OCT machines provide acquisition speeds of up to 100,000 depth-scans per second, enabling volume acquisition times of only a few seconds as well as the development of novel motion-based contrast mechanisms such as OCT angiography (OCTA) [26].

While the contrast of conventional OCT is based on the intensity of the light backscattered by the sample, functional OCT methods add information by more thoroughly analyzing the OCT signal, for instance spectroscopically or in terms of its polarization state. Some very popular approaches to functional OCT exploit the phase information already incorporated in the OCT signal. Phase data can be used to detect subtle relative displacements between successive scans. Such analysis enables the visualization of retinal perfusion by OCTA and quantitative blood flow measurements by Doppler OCT [27–29]. Phase-differences between consecutive depth profiles (A-scans) or tomograms (B-scans) can further be used to determine relative displacement and sample deformation in optical coherence elastography (OCE) [30–32]. Traditionally, in OCE a load is used to cause a deformation of the imaged sample and the measured pulse propagation is correlated to the impulse function to derive biomechanical properties such as stiffness or elastic modulus [31,32]. In ophthalmology, OCE was applied in the past to investigate various ocular structures ex vivo [33–36]. Recently, first demonstrations of in vivo OCE were reported for measuring pulse-induced deformations between the cornea and the retina, within the anterior chamber, as well as within the posterior eye [34,37–43]. Most recently, OCT based elastography was presented as a promising approach to measure pulsatile movements in the human optic nerve head in 2D [38,39] as well as to map relative pulsations between the retinal and scleral slab [44]. Here we present fundus elastography (FUEL) based on 4D-OCT for the quantitative assessment and depth-resolved mapping of pulsatile dynamics in the murine retina and choroid with high spatiotemporal resolution.

## 2. Methodology

### 2.1 OCT ophthalmoscope for 4D imaging of the posterior mouse eye

A homemade spectral domain polarization-sensitive OCT system was used to demonstrate the principle of depth-resolved deformation mapping with FUEL. Located in the animal facility of the Medical University of Vienna in close vicinity to the animal housing rooms, the setup was tailored for in-vivo imaging in the posterior eyes of small rodents. A detailed description of the instrument can be found elsewhere [45]. In brief, the optical layout was based on a broadband superluminescent diode ( λ = 840 nm, Δλ = 100 nm) providing an axial resolution of 3.8 μ m in eye tissue, a free-space Michelson interferometer with polarization optics, and a polarization-sensitive detection unit incorporating two identical spectrometers operating at a line rate of 83 kHz. The sample arm included an x-y galvanometer scanner and two telescopes optimized for retinal scanning in rodent eyes. BM-scans, i.e. 2,000 B-scan repeats at one position with a 1-mm lateral range, as well as volumetric scans spanning a retinal field of view of ∼ 1 mm ( x ) × 1 mm ( y ) sampled by 512 ( x ) × 400 ( y , with 5 repeats per position) × 1536 ( z ) voxels were acquired.

### 2.2 Processing pipeline

**Fig. 1.**
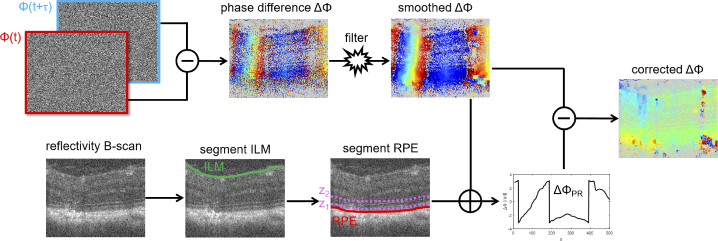
Data processing pipeline. Phase difference images ΔΦ were computed from subsequent B-scans and smoothed using a Gaussian convolution filter on complex phasors. The corresponding OCT reflectivity data was used to segment the inner limiting membrane (ILM) and the retinal pigment epithelium (RPE). The phase difference data in a slab around the photoreceptors, $\Delta \Phi_{PR}$ , was then subtracted from the smoothed phase difference image to reveal subtle displacements in the retina and choroid.

From the simultaneously recorded spectral interferograms, the complex OCT signals were computed for both polarization channels by standard spectral domain OCT processing including fixed pattern noise removal by background subtraction, linearization of the spectral data in wavenumber space, numerical dispersion compensation, and Fourier transformation. The intensity signals of both polarization channels as well as the phase signals of the co-polarized channel were used for the subsequent analysis schematically visualized in Fig. 1. In every B-scan, the inner limiting membrane (ILM) was segmented by detecting the signal surge at the vitreoretinal interface. In a next step, the retinal pigment epithelium (RPE) was segmented in the cross-polarized channel using the anterior edge of the strong signal caused by polarization scrambling due to melanin pigmentation. Global axial bulk motion was corrected using the ILM position as a reference for aligning the image data in a pixelwise fashion. In order to measure motion between consecutive B-frames, the phase difference between subsequent frames was calculated as

(1) $$\Delta \Phi =\text{arg}\left[e^{i[\Phi (x,z,t+\tau )-\Phi (x,z,t)]}\right]$$

where τ is the inter-frame time ( ∼ 7.7 ms). To reduce speckle noise and increase the signal-to-noise ratio (SNR) of phase difference signals, the phase difference images ΔΦ were used as the arguments of phasors weighted by the respective intensity images and smoothed in the x - z plane using a real-valued Gaussian convolution kernel G(x,z) with a standard deviation of 7 pixels ( ∼14μ m),

(2) $$\Delta \Phi_{smooth}(x,z,t)=\text{arg}\left[\sqrt{A(x,z,t)A(x,z,t+\tau )}e^{i\Delta \Phi (x,z,t)}⋆G(x,z)\right].$$

The average phase difference signal of a shallow band around the photoreceptor layer (PR) – i.e., anterior to the segmented RPE layer – was calculated as

(3) $$\Delta \Phi_{PR}(x,t)=\text{arg}\left[\sum_{z\prime =z_{RPE}+z_{1}}^{z_{RPE}+z_{2}}\sum_{x\prime =x-x_{1}}^{x+x_{1}}\sqrt{A(x\prime ,z\prime ,t)A(x\prime ,z\prime ,t+\tau )}e^{i\Delta \Phi (x\prime ,z\prime ,t)}\right].$$

where the dimensions of the averaging region were $x_{1}$ = 5 pixels ( ∼10μ m), $z_{1}$ = 5 pixels ( ∼10μ m), and $z_{2}$ = 35 pixels ( ∼66μ m). (Note that z is oriented in the posterior-anterior direction because the zero-delay was positioned just posterior to the sclera during eye alignment). For each frame, the above phase difference profile of the photoreceptor layer was used to subtract residual drifts in the phase difference B-scans caused by axial motion and also included subtle bulk motion that could not be removed by simply aligning the frames:

(4) $$\Delta \Phi_{rel}(x,z,t)=\text{arg}\left[e^{i[\Delta \Phi (x,z,t)-\Delta \Phi_{PR}(x,z,t)]}\right]$$

Since the posterior retina was used as a reference, subtle motion relative to the photoreceptor layer caused by deformation in the anterior retina as well as in the choroid and sclera could be visualized. The frame rate of ∼ 130 frames per second yielded an unambiguous measurement range of inter-frame displacements in the ±20μ m/s range. In order to increase the visibility of structural features, pixels with low SNR were masked in gray. Note that the two-step alignment process including the coarse frame-to-frame alignment (here using the ILM as a reference) and the fine alignment by subtracting the reference phase difference (here using the photoreceptors as reference) could be sped up by combining them in a single step using only one reference layer (e.g. only the photoreceptor layer).

In BM-scan data, the temporal evolution of $\Delta \Phi_{rel}(x,z,t)$ was investigated for several handpicked transverse positions ( x , z ) in different layers. The pulsatile frequency content of these temporal profiles was quantified by computing the fast Fourier transform (FFT) along the temporal axis, t , and charting the resulting spectral amplitude profiles.

To provide a quantitative readout of the pulsatile character which can be grasped at a glance, the absolute values of the BM-scan phase difference data of one pulse cycle were averaged as

(5) $$|\Delta \Phi_{rel}{|}_{avg}(x,z)=\frac{1}{N}\sum_{t=0}^{N\tau }|\Delta \Phi_{rel}(x,z,t)|$$

where N denotes the number of frames contained in one pulse cycle and was determined from the FFT of $\Delta \Phi_{rel}(x,z,t)$ .

While for the BM-scan data, the slow axis only encodes time ( y = 0), the galvanometer y-scanner was stepped along the slow axis for volumetric acquisitions. Because the heart rate of mice (482 ± 10 beats per minute [46]) is much faster than the chosen volume acquisition rate ( ∼ 3.9 per minute for 2,000 frames per volume) but much slower than the frame rate of our system ( ∼ 7,800 frames per minute), each volumetric OCT dataset contained 3D structural data modulated by the pulse with a pulse repetition rate of about 15 B-frames per heart cycle. Again, the actual number of frames per heart cycle, N , was determined for each volume from the fundamental spectral amplitude of the FFT of the phase difference data along the slow ( y , t ) axis. Then each volumetric dataset was decomposed into N ex post time-gated sub-volumes such that each of the N sub-volumes represented 1/N of the pulse cycle. The pulsatile characteristics were investigated for three slabs, namely (i) the anterior retina from the segmented ILM position until 30 pixels ( ∼57μ m) posterior to the ILM, (ii) the outer retina from 40 pixels ( ∼76μ m) to 10 pixels ( ∼19μ m) anterior to the segmented RPE, and (iii) the choroid from 5 pixels to 30 pixels posterior to the segmented RPE layer. In each slab, the phase difference data $\Delta \Phi_{rel}(x,z,t)$ was axially averaged,

(6) $$\Delta \Phi_{slab}(x,t)=\text{arg}\left[\sum_{z,\text{slab}}\sqrt{A(x,z,t)A(x,z,t+\tau )}e^{i\Delta \Phi_{rel}(x,z,t)}\right],$$

to generate a two-dimensional en-face map of the displacement characteristics. These maps were generated for each of the N sub-volumes representing the heart cycle, smoothed by a rolling median filter over 3 pixels in the y -direction, and mounted as a looped sequence in a movie. Finally, the signed as well as the absolute values of the phase difference data displacement maps $\Delta \Phi_{slab}(x,y)$ of each of the N sub-volumes were averaged to quantitatively assess the average net shift and the absolute shift in a slab over the heart cycle.

Data processing was performed in MATLAB (R2018b, MathWorks). Volume renderings were generated in Fiji [47].

### 2.3 Animals

The retinas of adult mice were investigated to prove the principle of 4D-OCT based in-vivo mapping of pulsatile tissue deformation dynamics. Imaging was performed under isoflurane anesthesia. Anesthesia was induced in a ventilated chamber for 4 minutes with isoflurane vaporized in oxygen at 4 % concentration. Thereafter, animals were placed in a comfortable 5-axis mount for imaging and were administered 2 % isoflurane via a nose cone. A heat pad was used to keep the animal warm. Tropicamide (5 mg/mL, Agepha Pharma s.r.o., Senec, Slovakia) was used to dilate the pupil and artificial tear drops (Oculotect, Thea Pharma GmbH, Vienna, Austria) were frequently applied to keep the corneas of both eyes moist. Wild type mice with a B6SJL background (The Jackson Laboratory, Bar Harbor, USA) were investigated, as well as a very low density lipoprotein receptor (VLDLR) knockout mouse model (B6;129S7- $Vldlr^{tm1Her}$ /J, The Jackson Laboratory, Bar Harbor, USA [48]), which develops neovascular lesions in the retina similar to retinal angiomatous proliferation in exudative AMD [49]. Animal protocols were approved by the animal ethics committee of the Medical University of Vienna and the Austrian Ministry of Education, Science, and Research (BMBWF/66.009/0272-V/3b/2019).

## 3. Results

### 3.1 Tomographic imaging of pulsatile tissue displacements

**Fig. 2.**
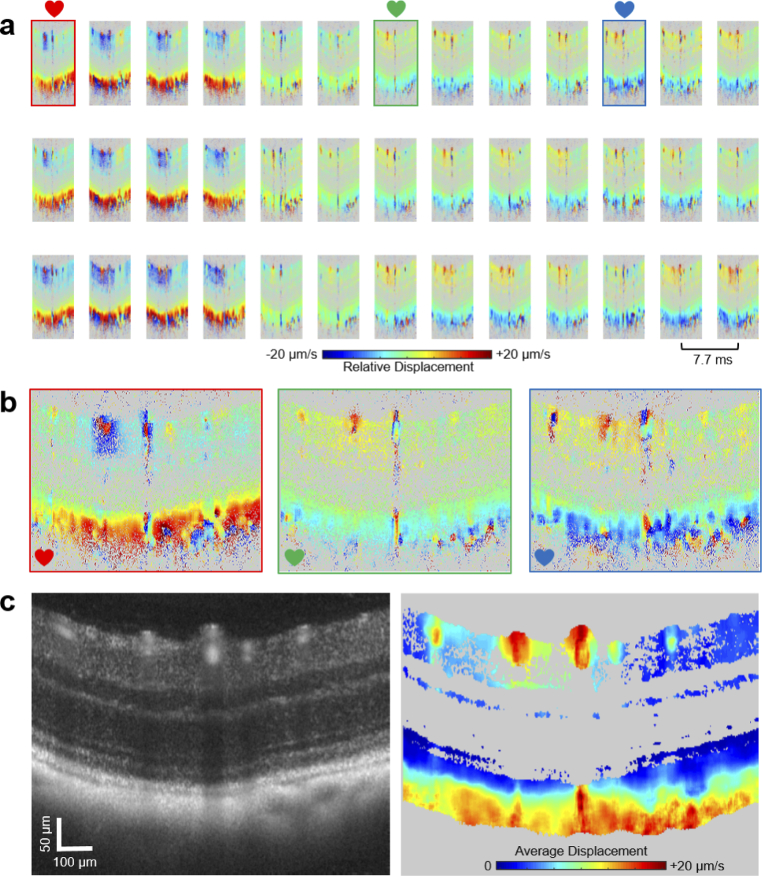
Cross-sectional imaging of pulsatile tissue displacements in a wildtype mouse retina. (a) Sequence of phase difference images over three heart cycles reveal repeated patterns of pulsatile motion and deformation in the vicinity of retinal and choroidal vessels. (b) Magnified view of three phase difference images at different phases of the cardiac cycle as indicated by the little colored hearts in panel (a). Note the strong displacements localized in the surrounding of retinal vessels and the rather uniformly strong relative shifts in and beneath the choroid. (c) Reflectivity B-scan image averaged over one pulse cycle (left). B-scan image mapping the average displacement, i.e. the average phase shift magnitudes, across the retina and choroid.

A sequence of B-scans revealing the pulsatile dynamics in the retina of a wildtype mouse is shown in Fig. 2. Phase difference images from three consecutive heart cycles are arrayed in Fig. 2(a) and illustrate the repetitive characteristic of the deformation dynamics relative to the photoreceptor layer. In these images, the negative (blue) and positive (red) halves of the color scale represent relative motion in posterior and anterior direction, respectively. In the vicinity of large retinal vessels, pronounced deformation patterns were observed. While pulsatile deformations were rather localized around the vessels in the retina and tapered off in their surroundings, the densely vascularized choroid presented a similarly strong but spatially uniform displacement characteristic. Figure 2(b) provides a magnified view of three phase difference images marked by colored frames in Fig. 2(a). These images showcase the deformation differences during the pulsatile cycle. Moreover, the oppositely directed orientation of the displacements in retina and choroid can be observed with respect to the photoreceptor layer, which acted as the reference position. Due to the flow of blood through large retinal vessels, shadows of artificially elevated deformation were observed in the posterior retina and choroid. Note that due to the smoothing operation on the phase difference images, spatially confined motion (e.g. in retinal capillaries) was washed out. Figure 2(c) shows a reflectivity image alongside an image representing the average absolute displacement over one pulse cycle for each image pixel. Here, similar to a power Doppler representation, the spatial differences of the retinal and choroidal pulsation amplitude patterns become apparent.

### 3.2 Quantitative charting of pulsatile motion with high 3D resolution

**Fig. 3.**
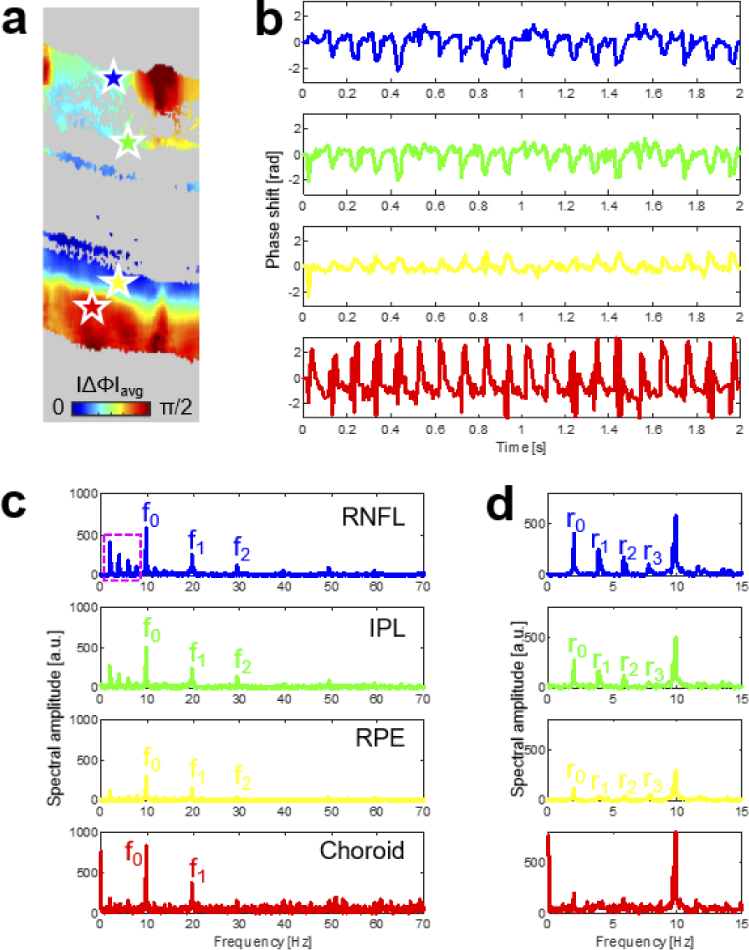
Time-frequency analysis of localized pulsatile displacements in the retina and choroid. (a) Cropped average displacement B-scan image with colored stars indicating measurement locations in the retina and choroid. (b) Pulsatile profiles in the retinal nerve fiber layer (RNFL), inner plexiform layer (IPL), retinal pigment epithelium (RPE), and choroid. (c) Spectral amplitudes of the phase difference sequences shown in panel (b). All layers present similar fundamental frequencies of ${\text{f}}_{0}$ = 9.9 Hz and harmonics ( ${\text{f}}_{1}$ , ${\text{f}}_{2}$ ) related to pulsatility. In the anterior retina, deterministic low frequency content is also visible (see pink square in the top plot). (d) Zoom-in on the low frequency range of the spectral amplitudes visualizes a peak at ${\text{r}}_{0}$ = 1.95 Hz as well as its first three harmonics.

The BM-scan phase difference data can be used to analyze the pulsation characteristics in each transverse image pixel (*x,z*). Results from a time-frequency analysis of localized pulsatile displacements in the retina and choroid from the dataset qualitatively presented in Fig. 2 are shown in Fig. 3. Figure 3(a) indicates four representative locations in the retinal nerve fiber layer (RNFL), inner plexiform layer (IPL), RPE, and choroid that were chosen for spatiotemporal measurements. The corresponding pulsatile phase difference profiles across the retina and choroid as well as the spectral amplitudes of their FFTs are shown in Fig. 3(b) and Fig. 3(c), respectively. Here, similar fundamental frequencies of 9.9 Hz and harmonics related to pulsatility were observed in all four locations, albeit with different spectral intensity. In addition, some deterministic low frequency content peaking at 1.95 Hz and up to three harmonics were visible in the anterior retina (see Fig. 3(d)).

### 3.3 Time gated en-face maps of fundus pulsatility over the heart cycle in a control mouse

**Fig. 4.**
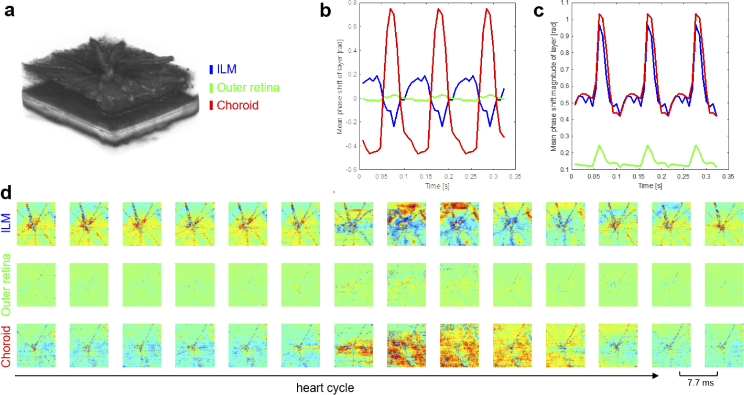
Time-gated imaging of retinal and choroidal pulsations in a wildtype mouse by 4D-OCT. (a) Volume rendering of the reflectivity image data indicating the investigated slabs in the ILM, outer retina and choroid. (b) Temporal evolution of the relative phase shift averaged across each of the three slabs. (c) Magnitude of the pulsatile displacements computed as the average absolute shift within each slab. (d) Spatiotemporal mapping of fundus pulsations in ILM, outer retina and choroid over one heart cycle. Visualization 1 provides a loop of the pulsation maps.

Figure 4 shows retinal and choroidal pulsation results from a time-gated analysis of 4D-OCT image data from a wildtype mouse. The three analyzed slabs are indicated next to a volume rendering of the reflectivity image data in Fig. 4(a). The temporal evolution of the relative phase shift averaged within each of the three slabs – the ILM, outer retina and choroid – is charted in Fig. 4(b). In contrast to the strong average displacement in the choroid, the mean phase shift in the retina was much weaker, in particular in the outer retina. In Fig. 4(c), plots of the magnitude of the pulsatile displacements computed as the average absolute shift within each slab are shown. A clear pulsatile characteristic can be observed in all three slabs. Interestingly, the average pulsation magnitude at the vitreoretinal interface and in the choroid are quite similar. The time-gated sequence of the displacement en-face images is displayed in a tabular form in Fig. 4(d). Here, the spatiotemporal variations of fundus pulsations in the ILM, outer retina and choroid over one heart cycle can be observed. The propagation of the tissue deformation waves can be seen in Visualization 1 which provides a looped visualization of the pulsation maps from the same dataset.

### 3.4 Time gated en-face maps of fundus pulsatility over the heart cycle in a VLDLR mouse model

**Fig. 5.**
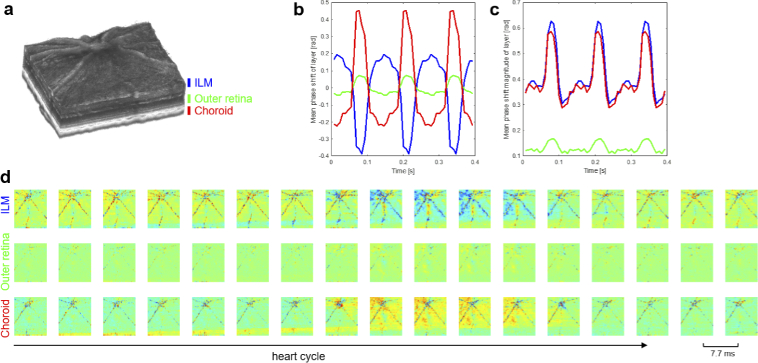
Time-gated imaging of retinal and choroidal pulsations in a VLDLR mouse by 4D-OCT. (a) Volume rendering of the reflectivity image data indicating the investigated slabs in the ILM, outer retina and choroid. (b) Temporal evolution of the relative phase shift averaged across each of the three slabs. (c) Magnitude of the pulsatile displacements computed as the average absolute shift within each slab. (d) Spatiotemporal mapping of fundus pulsations in ILM, outer retina and choroid over one heart cycle. Visualization 2 provides a loop of the pulsation maps.

**Fig. 6.**
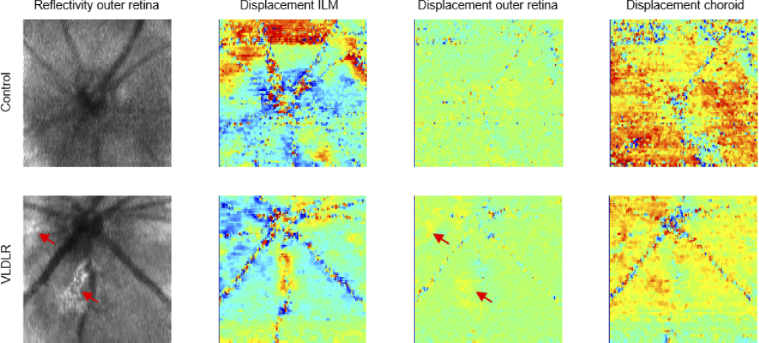
Reflectivity and pulsatile displacements in en-face maps of a control mouse (top row) and a VLDLR mouse retina (bottom row). The phase difference maps represent the displacement patterns at the peak of the pulsatile cycle. The patches of increased pulsatility in the outer retina of the VLDLR mouse coincide with hyperscattering regions in the same layer (red arrows).

Time-gated displacement images of retinal and choroidal pulsations in a VLDLR mouse reconstructed for three different en-face slabs are shown Fig. 5. The reflectivity volume rendering in Fig. 5(a) visualizes the locations of the investigated slabs in the ILM, outer retina and choroid. In contrast to the corresponding control mouse data, the temporal evolution of the mean relative phase shifts of ILM and choroid appeared to be of similar amplitude (Fig. 5(b)). However, they were similarly oriented in opposite directions and of greater amplitude than the pulsation profile of the outer retina. It is worth noting that the scan locations – centered at the ONH vs. slightly inferior to the ONH – were not exactly the same in Figs. 4 and 5. The magnitude of the pulsatile displacements i.e. the average absolute shift within each slab, is plotted in Fig. 5(c) and exhibits similar characteristics to those in Fig. 4(c). In Fig. 5(d), sequences of en-face maps visualize the effect of fundus pulsations in ILM, outer retina and choroid. A movie of the looped sequences is shown in Visualization 2. Since in the original data set consisting of 2,000 frames the heart cycle covered ∼ 17 frames in this mouse eye, each en-face map consists of 117 frames in the y direction. Notably, in some regions of the outer retina, stronger pulsatile displacements were observed for frames 9 through 12. A comparison of the frames at the peak of the pulsatile displacements is shown in Fig. 6. The patches of increased pulsatility in the outer retina coincided with hyperscattering regions in the same layer, which have been associated with neovascular lesions in our earlier work [50,51].

## 4. Discussion

The analysis of the phase of OCT signals enables quantitative measurements of subtle, sub-wavelength scale displacements between acquisitions at different time points. While OCT phase analysis approaches have been widely exploited for measuring retinal blood flow using Doppler OCT and the visualization of retinal microvasculature using OCTA [27–29], phase-based tissue displacement measurements – often referred to as optical coherence elastography [31] – have only been started to be explored. Phase-based analysis in BM-scans was first presented to visualize pulsatile motion of the ONH [38,39], and later used to investigate thermal expansion of retinal tissue [52,53]. Using full-field OCT, pulse wave propagation of single vessels was imaged in small fundus patches by analyzing relative motion of the retinal nerve fiber layer (RNFL) with respect to the retinal pigment epithelium (RPE) [54]. More recently, we have demonstrated the visualization of pulsatile motion between the retinal and chorioscleral slab in the rat retina in vivo [44]. This work has taken this analysis approach to the next level by introducing 4D imaging of pulsatile tissue displacements with high spatiotemporal resolution in the murine retina and sclera.

**Fig. 7.**
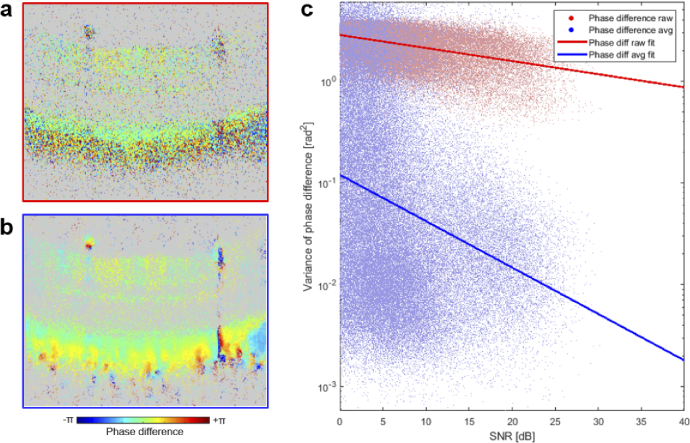
Effect of local averaging on phase difference image data. (a) Representative frame before averaging. Pixels with SNR < 6 dB in the corresponding reflectivity frame were masked in gray. (b) Same frame after smoothing using a Gaussian kernel as described in Eq. (2). (c) Plot of phase variance vs. SNR for each image pixel in panels (a) and (b) are shown in red and blue color, respectively. Linear fits of the respective (logarithmic) data indicate the improvement of phase variance as the SNR increases.

In our approach, the temporal resolution was determined by the B-scan rate and on the order of 7.7 ms. For the analysis of pulsatile motion in mice under isoflurane anesthesia presented here, the pulse wave was sampled at 14-17 time points per cycle, which enabled a good coverage of pulsatile motion. Note that in humans, which have a ∼ 5 times lower heart rate, the sampling density per heart cycle would be much higher for the same B-frame rate. However this increased sampling density would come at the cost of covering less heart cycles within the same acquisition time, which is rather long and therefore might not be feasible for humans. The spatial resolution of our mouse ophthalmoscope is on the order of a few microns in x , y and z ; however due to the Gaussian smoothing operation on the phase difference data (Eq. (2)), the point spread functions in axial and transverse direction were broadened by the convolution with the 14- μ m Gaussian kernel. While the smoothing step decreased the spatial resolution to an extent that it obstructed the analysis of small features such as retinal capillaries and their surroundings, the qualitative and quantitative assessment of the phase difference image data greatly improved by filtering. On one hand, the speckle noise was suppressed by the smoothing operation; on the other hand, the variance of local phase difference measurements was massively improved by the averaging within the Gaussian kernel. To further substantiate this point, we show the same phase difference frame before and after Gaussian filtering of the complex phasors in Fig. 7(a) and (b), respectively. The same thresholding mask was applied such that pixels with an SNR < 6 dB in the reflectivity channel were set to gray. The clearly reduced noise in the phase difference image is obvious and can further be observed in Fig. 7(c) where for each image pixel the phase variance in a 10 × 10-pixel neighborhood was plotted as a function of SNR. Note that the phase variance levels depend on SNR [55,56]. Still (and not surprisingly), the phase variance values observed in real-world retinal imaging data are much worse than phase variance measurements performed using a mirror with very high SNR as a sample to estimate phase stability (specifically, 5 × ${\mathrm{10}}^{-3}$ ${\mathrm{rad}}^{2}$ at SNR = 32.5 dB for our setup). This suggests that it is worthwhile to estimate phase variance of the actual image data in addition to a (high-SNR) specification measurement of a system’s phase stability.

The volume scan pattern used for the 4D-OCT data sets shown in Figs. 4–6 was originally designed for the acquisition of OCT angiography (OCTA) data. It comprised a grid of 400 y -positions and five repeats per *y*-position. This rigid pattern is not ideal for the proposed measurement approach as the data acquired during one heart cycle are spread over ∼ 15 frames and thus over several y -positions. As the transverse resolution achieved in the actual OCT data shown in this manuscript was worse than theoretically expected, we observed that the speckles were still overlapping up to 15 frames apart and allowed to use the proposed phase analysis. Still, both the SNR of the phase difference data and the visualization of small structures would definitely benefit from the use of a more sophisticated scan pattern. One potential improvement would be the use of a stepped scan pattern that is synchronized to the pulse frequency and only steps to the next y -position when one pulse cycle has completed. The synchronization could be realized in real-time by using an external pulse measurement such as plethysmography (e.g. using a tail cuff sensor for rodents, or an ear clip sensor for human use). An alternative approach could be real-time processing of the OCT data and performing a short-time Fourier transform in y -direction to assess the pulsation waveform and frequency in the actual OCT data and swiftly updating the galvo waveform. Both approaches would also resolve a potential desynchronization issue caused by a drift of the heart rate during the volumetric acquisition. The 4D scan method would definitely become more robust by implementing one of the aforementioned synchronization approaches.

Blood flow in the retinal vasculature produces multiple scattering trails casted over structures in the posterior layers and choroid. These trails are caused by forward multiple scattering [57] and often referred to as OCT projection artifact [58]. Different methods have been devised to mitigate the artificial flow signal produced in OCTA images of the posterior retina and choroid [58]. Also the FUEL images presented in Figs. 2–6 reveal similar projection artifacts beneath large retinal vessels (see for instance the choroid beneath the retinal vessel in the center of Fig. 2(b) and the imprint of the retinal vessels onto the choroidal displacement maps in Fig. 6). While it may be possible to computationally remove the impact of the retinal signals trails on the choroid to some extent, an easy solution allowing for a clearer visualization of pulsatile displacements in the posterior layers would be to simply mask these pixels (i.e. set them to background level) and discard their data from any quantitative analysis.

This work introduced an approach to visualize and quantitatively assess pulsatile motion in the retina and choroid and, in our opinion, impressively demonstrated the feasibility and capabilities of 4D-OCT to map deformations volumetrically, non-invasively, and in vivo. However, using the common 840-nm band for spectral domain OCT, the penetration beyond the highly pigmented mouse choroid is very low. The use of high-speed OCT at 1060 nm or even 1310 nm may enable deeper penetration into the sclera [59–61], which could be particularly interesting for studying the relation of tissue biomechanics and intraocular pressure in the papilla region (in particular the lamina cribrosa) in vivo and with high resolution. In addition, the longer imaging range and shallow SNR roll-off of swept source OCT would enable simultaneous imaging of the cornea and the posterior eye and thus enable more traditional fundus pulsation measurements based on the relative motion across the entire eye ball – yet in a volumetric fashion.

One challenge during our experiments was respiratory motion which, at least for OCT imaging in the small mouse eye, can introduce artifacts during data acquisition by displacing the beam on the pupil and therefore offsetting the scanned position in the posterior eye. Figure 3 shows the presence of a low-frequency component in the order of magnitude of the respiration rate of mice. While motion artifacts may be avoided or at least reduced by restraining the animal more tightly or by using sophisticated hardware additions such as retinal or pupil tracking [62,63], it might also be possible to remove some of these artifacts in post-processing. For instance, high-pass Fourier filtering could be used to suppress low-frequency artifacts.

We demonstrated imaging in a wildtype mouse and in a mouse model of retinal neovascularization which had developed lesions at several locations within the scanned field of view. Of note, the pulsatile characteristics at the lesion site exhibited a stronger displacement compared to the lesion-free environment and also to the outer retina of the control mouse (cf. Figure 6). As this is an interesting observation that deserves a deeper investigation with sufficient statistical power, a fruitful next step will be an experiment in a larger cohort of knockout and control mice. In particular, a longitudinal study of the pulsatile characteristics at lesions sites and under various physiologic conditions may be an exciting target. Moreover, this approach may not only be applied to mouse models of neovascular retinal degeneration but also to rodent models of other diseases with potential alterations of tissue biomechanics and/or pulsatility. Ultimately, our approach could be adopted for imaging in human eyes, e.g. by implementing proper scan protocols with time-gated acquisitions. Still, as the method presented in this paper is derived from a standard OCTA scan protocol with some alternative data processing, it has the potential to be applied to millions of OCTA datasets harbored at OCT machines at ophthalmology clinics worldwide – even retrospectively.

## 5. Conclusion

In this work, we presented 4D mapping of pulsatile motion in the mouse retina by analyzing the phase difference between subsequently acquired B-scans and using the signal of the photoreceptor layer as a reference. With a frame rate of 130 Hz, displacements in the ±20μ m/s range were measured. Sequential imaging of pulsatile dynamics was performed using a BM-scan protocol and a volume scan protocol. In vivo imaging in the eyes of a wildtype mouse eye and a VLDLR knockout mouse revealed pulsatile displacements localized in the vicinity of retinal vessels as well as in the choroid. In the outer retina of the VLDLR mouse, increased pulsatility was observed at locations exhibiting high reflectivity associated with neovascular lesions. Our results prove the concept of fundus elastography (FUEL) based on high-speed 4D-OCT imaging is capable of visualizing subtle tissue deformation dynamics related to ocular pulsation. This approach holds promise for a powerful retinal elastography technique that may enable a new paradigm of OCT based measurements and image contrast.

## Data Availability

Data underlying the results presented in this paper are not publicly available at this time but may be obtained from the authors upon reasonable request.
